# Harnessing the Immune System to Fight Multiple Myeloma

**DOI:** 10.3390/cancers13184546

**Published:** 2021-09-10

**Authors:** Jakub Krejcik, Mike Bogetofte Barnkob, Charlotte Guldborg Nyvold, Thomas Stauffer Larsen, Torben Barington, Niels Abildgaard

**Affiliations:** 1 Centre for Cellular Immunotherapy of Haematological Cancer Odense (CITCO), Odense University Hospital, 5000 Odense, Denmark; jakub.krejcik@rsyd.dk (J.K.); Mike.Bogetofte.Barnkob@rsyd.dk (M.B.B.); Charlotte.Guldborg.Nyvold@rsyd.dk (C.G.N.); Thomas.Stauffer.Larsen@rsyd.dk (T.S.L.); Torben.Barington@rsyd.dk (T.B.); 2 Department of Haematology, Odense University Hospital, 5000 Odense, Denmark; 3 Haematology Research Unit, Department of Clinical Research, University of Southern Denmark, 5000 Odense, Denmark; 4 Department of Clinical Immunology, Odense University Hospital, 5000 Odense, Denmark; 5 Haematology-Pathology Research Laboratory, Research Unit for Haematology and Research Unit for Pathology, University of Southern Denmark and Odense University Hospital, 5000 Odense, Denmark

**Keywords:** multiple myeloma, immunotherapy, immune modulation, allogeneic stem cell transplantation, cancer vaccination, adoptive cell transfer, monoclonal antibodies, immunomodulatory drugs, immunogenic cell death

## Abstract

**Simple Summary:**

Multiple myeloma treatment has developed enormously within the last two decades. Most recently immunotherapies have been introduced. Monoclonal antibodies targeting the plasma cell surface marker CD38, daratumumab and isatuximab, have revolutionized the standard of care treatment, and CAR T-cell therapy has been FDA-approved for the treatment of relapsed, refractory multiple myeloma. However, many other immunotherapeutic principles are under current clinical testing. It is well described that immune dysfunction is present in multiple myeloma and worsens by disease progression and may even be involved in the transformation to malignancy from benign precursor states, smoldering myeloma, and MGUS. Thus, attempts to revive and engage the innate and adaptive immune system are appealing. The ultimate goal is the cure or prevention of cancer development. In this review, the reader receives basic information on the immune dysfunction in multiple myeloma, a thorough summary of the ways to harness the immune system in treatment, the current status of clinical development, and future aspects.

**Abstract:**

Multiple myeloma (MM) is a heterogeneous plasma cell malignancy differing substantially in clinical behavior, prognosis, and response to treatment. With the advent of novel therapies, many patients achieve long-lasting remissions, but some experience aggressive and treatment refractory relapses. So far, MM is considered incurable. Myeloma pathogenesis can broadly be explained by two interacting mechanisms, intraclonal evolution of cancer cells and development of an immunosuppressive tumor microenvironment. Failures in isotype class switching and somatic hypermutations result in the neoplastic transformation typical of MM and other B cell malignancies. Interestingly, although genetic alterations occur and evolve over time, they are also present in premalignant stages, which never progress to MM, suggesting that genetic mutations are necessary but not sufficient for myeloma transformation. Changes in composition and function of the immune cells are associated with loss of effective immune surveillance, which might represent another mechanism driving malignant transformation. During the last decade, the traditional view on myeloma treatment has changed dramatically. It is increasingly evident that treatment strategies solely based on targeting intrinsic properties of myeloma cells are insufficient. Lately, approaches that redirect the cells of the otherwise suppressed immune system to take control over myeloma have emerged. Evidence of utility of this principle was initially established by the observation of the graft-versus-myeloma effect in allogeneic stem cell-transplanted patients. A variety of new strategies to harness both innate and antigen-specific immunity against MM have recently been developed and intensively tested in clinical trials. This review aims to give readers a basic understanding of how the immune system can be engaged to treat MM, to summarize the main immunotherapeutic modalities, their current role in clinical care, and future prospects.

## 1. Introduction

**Figure 1:**
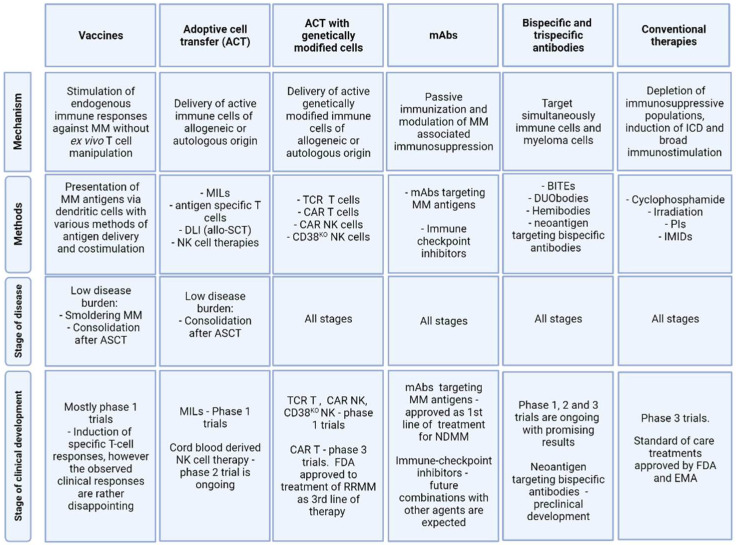
Overview of immunotherapeutic principles in Multiple Myeloma. ASCT: Autologous stem cell transplantation; DLI: Donor lymphocyte infusion; ICD: Immunologic cell death; IMIDs: Immunomodulatory drugs; mAbs: Monoclonal antibodies; MILs: Marrow infiltrating lymphocytes; PIs: Proteasome inhibitors.

Multiple myeloma (MM) is a very heterogeneous plasma cell cancer that differs extensively in clinical behavior, prognosis, and response to treatment [1]. MM is almost always preceded by a premalignant stage called monoclonal gammopathy of undetermined significance (MGUS) [2]. Genetic data from MGUS patients have provided evidence that clonal plasma cells already have a genetically advanced landscape at this stage, wherein various clones coexist and carry the same genetic changes as found in malignant MM cells [3,4,5]. In spite of this genomic complexity, most of MGUS cases are clinically stable, and never progress into MM [6]. Interestingly, animal studies have shown that MGUS cells mediate progressive growth upon xenotransplantation in humanized mice and several other studies have demonstrated the capacity of innate and adaptive immune cells to recognize MM/MGUS cells and potentially mediate the control of tumor growth [7,8]. This suggests that the growth rate and clonal evolution of MGUS cells into MM may depend in part on extrinsic factors as for instance immunosurveillance [3,9]. Importantly, the immune system in MM patients demonstrates progressive impairment compared to MGUS stages [10,11,12]. Several mechanisms have been proposed to be involved in the fading tumor surveillance that may be involved in disease progression, including production of immune-suppressive cytokines, suppression mediated by regulatory T-cells [13,14,15,16], myeloid-derived suppressor cells [17], tumor-associated macrophages [18,19], and the expression of inhibitory immune checkpoints on cells present in the tumor microenvironment (TME) [20,21,22]. Another common feature of disease progression appears to be a suppression of T-cell activity by an increase in ectoenzymatic activity in hypoxic TME. A recent study demonstrated that immunosuppressive adenosine generated in the bone marrow niche through a CD38-mediated pathway correlates with the progression of MM [23,24]. In addition to adaptive T-cell mechanisms, innate immunity also plays a role in immune-mediated control of MM. In particular, the importance of NK cells in MM control has been demonstrated in several studies [25,26,27]. Accordingly, NK cells from MM patients show both quantitative and qualitative changes compared to NK cells from healthy donors [28,29]. Taken together, this evidence paints a complex picture where progressive immunosuppression associates with MM development. Interactions between tumor plasma cells and the bone marrow (BM) microenvironment contribute to generating an immunosuppressive milieu characterized by a high concentration of immunosuppressive factors, loss of effective antigen presentation, effector cell dysfunction, and expansion of immunosuppressive cell populations [30]. Strategies aimed at increasing immune surveillance and mitigating immunosuppression may have important therapeutic implications in MM, especially for long-term control and potential cure. This article provides an overview of methods (Figure 1) that can be used to harness the immune system in the fight against MM and put them into the context of current treatment algorithms.

## 2. T Cell and NK Cell-Dependent Therapies without Genetic Manipulation (Allogeneic Stem Cell Transplantation, Vaccination Strategies)

### 2.1. Allogeneic Stem Cell Transplantation (Allo-SCT)

**Figure 2:**
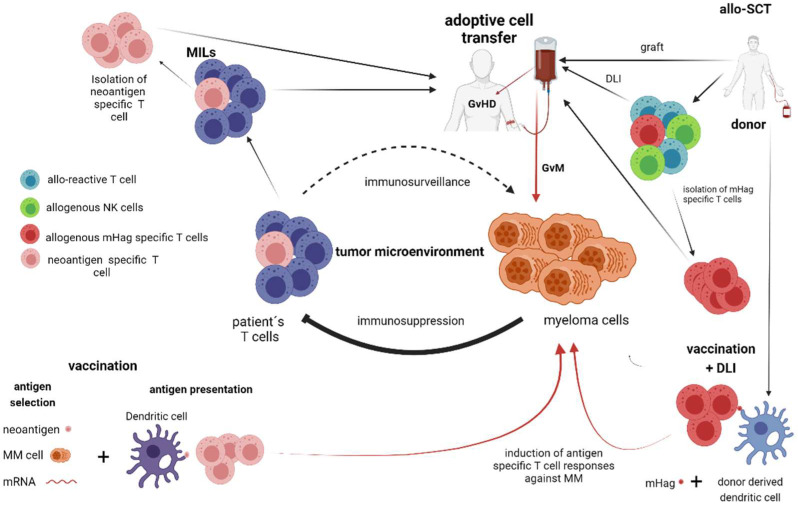
T-cell and NK-cell-dependent therapies without genetic manipulation (allogeneic stem cell transplantation, vaccination strategies, adoptive immune transfer of lymphocytes without genetic manipulation). Allo-SCT: Allogeneic stem cell transplantation; GvM: Graft versus myeloma; IMIDs: immunomodulatory drugs; MILs: Marrow-infiltrating lymphocytes; MDSCs: Myeloid-derived suppressor cells; TAMs: Tumor-associated macrophages; Tregs: Regulatory T cells; mHag: Minor histocompatibility antigen.

Allo-SCT provided the first evidence that MM can be cured by harnessing the immune system [31,32]. Its therapeutic effect, the graft-versus-myeloma (GvM) effect, is based mainly on an alloimmune response of donor T-cells directed against tumor cells. Donor T-cells attacking the tumor cells (Figure 2) are most frequently directed at host (allo)-specific antigens rather than tumor-specific antigens [32]. Therefore, GvM is often accompanied by graft-versus-host disease (GvHD) which needs to be prevented and treated by immunosuppression. GvHD and opportunistic, life-threatening infections cause high non-relapse mortality in allo-transplanted patients. However, it should be recognized that allo-SCT offers a potential cure in a carefully selected subgroup of high-risk MM patients with acceptable toxicity and preserved quality of life [31]. Interestingly, even in HLA-matched transplant settings, the T-cell alloreactivity leading to both GvM and GvHD exists. In these cases, alloreactivity might be caused by T-cell recognition of minor histocompatibility antigens (mHags), polymorphic HLA-bound peptides encoded by genes that differ between donor and recipient [33,34]. Variations in intracellular proteins that exist between the donor and recipient are due to the evolutionary occurrence of single nucleotide polymorphisms (SNPs) in the coding regions of the genome, creating peptides that differ in their amino acid sequence [34]. There have been identified mHags expressed in a hematopoietic-specific manner only, which represent a promising target for immunotherapy of hematological malignancies [35]. Various immunotherapeutic strategies using tumor-specific mHag-directed T-cell responses are under clinical development. These include mainly adoptive T-cell transfer with ex vivo generated mHag-specific T-cells and mHag-loaded dendritic cell (DC) vaccination [36,37,38]. The major limitation for the application of mHag-specific therapy is that donor and recipient need to express the appropriate HLA molecule for presentation of the specific mHag epitope and thus only a small group of patients can be treated with each mHag. With the advances of genomics, novel strategies for the identification of mHags have recently been introduced, which might broaden the applicability of this method in the near future [35].

### 2.2. Vaccination Strategies

MM vaccination strategies aim to stimulate endogenous immune responses against MM without ex vivo T-cell manipulation (Figure 2). Vaccines have the greatest potential in the prevention of disease progression and, therefore, most vaccine strategies have been tested post autologous stem cell transplantation (post-ASCT) or in smoldering myeloma, where tumor burden is low [39]. Key aspects of the vaccination approach are antigen selection, method of immune presentation, mechanisms to enhance immune responses, e.g., by co-medication, and finally to choose the stage of disease for treatment. Many vaccination strategies use dendritic cell presentation of tumor antigens. Autologous cancer cells themselves can serve as an antigen source, which have the potential to stimulate multiplex immunity to known cancer antigens, patient-specific neoantigens, but also previously unidentified antigens. In this approach, autologous cancer cells are physically fused with the dendritic cells [40]. Such a fusion vaccine was employed in a phase 2 clinical trial where the vaccine was administered post-ASCT. Remarkably, 24% of patients who achieved a partial response following transplant were converted to CR/nCR after vaccination [40]. Other routinely used dendritic-cell–based approaches are electroporation of dendritic cells with messenger RNAs encoding the myeloma antigens [41] or differentiation of dendritic cells ex vivo in the presence of tumor antigens (peptide loading) [42]. Granulocyte-macrophage colony-stimulating factor (GM-CSF) has been extensively investigated as a vaccine adjuvant, based on its ability to recruit and activate dendritic cells in vivo [39,43]. GVAX is a vaccine platform in which cells from two myeloma cell lines are admixed with K562 cells modified to express GM-CSF. Durable PFS and emergence of myeloma-specific immune responses were reported in stable patients with low levels of detectable MM on lenalidomide-containing regimens who received GVAX vaccination [44]. GVAX vaccine is currently evaluated in a phase II trial for MM with a CR/nCR after initial therapy. The goal of this trial is to extend remissions and potentially deepen responses to a minimal residual disease (MRD) negative state (NCT03376477).

The selection of an appropriate antigen is critical for the development of a vaccine strategy that preserves tumor specificity and immunologic efficacy. Cancer/testis (CT) antigens (e.g., NY-ESO-1 and SP17), a group of tumor antigens with normal expression restricted to male germ cells and pathological expression in malignant plasma cells, are frequently used and tested in diverse clinical trials with different designs of vaccines [45]. Interestingly, hypomethylating agents routinely used in hematological praxis have the capacity to increase the expression of CT antigens and their combination with vaccination against these antigens might be beneficial [46]. Vaccines against normal peptides preferentially expressed by MM cells represent another approach. The vaccine PVX-410, targeting XBP1, CD138, and CS1, has been developed and tested in smoldering myeloma with or without the addition of lenalidomide. Immune responses were observed, as indicated by an increase in the percentage of tetramer-positive cells and interferon γ–positive cells, which was further enhanced in combination with lenalidomide [47].

With the advances of genomics, tumor-specific mutations (neoantigens) are becoming specific targets for cancer immunotherapy [48,49,50]. Importantly, a recent study has demonstrated immunogenic neoantigens in MM, which were able to elicit T-cell-specific responses against malignant plasma cells. In addition, shared neoantigens, predicted in oncogenic driver genes, have been identified and it has been proposed that they could be targeted by “off-the-shelf” immunotherapeutic approaches including vaccination [51]. Currently, only one study vaccinating against patient-specific neoantigens (NCT03631043) is ongoing in smoldering MM. In an allo-SCT setting, donor-derived dendritic cells loaded with mHags followed by DLI are capable of inducing objective mHag-specific T-cell responses. However, the observed clinical responses are disappointing, indicating the need for improvements of the current vaccination strategy [37].

There are many challenges in vaccine approaches, including the dysfunctional immune system of MM patients and the inability to generate large numbers of antitumor cells with high affinity for tumor antigens, as well as an immunosuppressive TME that can suppress T-cell effector mechanisms [52]. Current research focuses on further improvements of the vaccination strategy, toward translating the observed T-cell responses induced by vaccination into more robust clinical responses. Many of the difficulties of optimizing vaccination strategies might be overcome by the use of adoptive cell transfer of antitumor immune cells which is further discussed below.

## 3. Adaptive Immune Transfer of Autologous and Allogeneic Lymphocytes without Genetic Manipulation—(Marrow-Infiltrating Lymphocytes, Donor Lymphocyte Infusions, Adoptive Transfer of NK-Cells)

### 3.1. Marrow-Infiltrating Lymphocytes (MILs)

The ability of expanded tumor-infiltrating lymphocytes (TIL) to induce durable clinical remissions in melanoma patients was one of the first demonstrations of the therapeutic potential of lymphocytes isolated from tumor sites [53]. Myeloma-specific T-cells isolated from the BM of patients with progressive myeloma can generate strong, tumor-specific cytolytic responses to autologous, tumor-loaded dendritic cells [54]. In another study, MILs were also able to target both the terminally differentiated CD138+ plasma cells and the myeloma precursors as was shown by a profound inhibition in a tumor clonogenic assay [55]. The antigens that mediated the recognition of clonal cells in these studies have not been identified. Several recent studies have revealed the critical role of neoantigen-specific T-cells in maintaining durable responses following adoptive cell transfer in epithelial cancer [56,57,58]. Importantly, MM, compared to epithelial cancers and melanoma, is a malignancy with a lower mutational burden and thereby a lower likelihood of neoantigen-specific T-cells being present in the TME [59,60]. However, a recent study has demonstrated that neoantigens in MM are immunogenic and can elicit neoantigen-specific T-cell responses against malignant plasma cells [51]. The whole bulk of isolated and ex vivo expanded MILs from patients (Figure 2) can be safely re-infused as cellular therapy. Such an approach was evaluated in a phase 1 clinical trial where MILs were expanded ex vivo and subsequently infused after ASCT as a part of consolidation. This approach induced durable myeloma-specific immune responses that correlated with superior clinical outcomes [61].

More broadly in cancer, the intrinsic capacity of intratumoral T-cells to recognize adjacent tumor tissue seems to be variable [62]. Strategies expanding specifically neoantigen-specific T-cells (Figure 2) have been described and therapeutically used in malignancies other than MM [49,58,59]. There is increasing research attention towards identifying and selecting neoantigen-specific T-cells for therapeutic purposes. However, such a “precise targeting” strategy poses a great challenge in terms of the identification and isolation of these cells. To this end, various methods have been proposed and developed [63].

### 3.2. Donor Lymphocyte Infusions (DLI)

DLI is the adoptive transfer of donor lymphocytes to the allogeneic setting and represents an important treatment strategy for patients with hematologic malignancies who have experienced relapse after allogeneic stem-cell transplantation (Allo-SCT) [64]. Nevertheless, the role of donor lymphocyte infusion (DLI) in the treatment of MM is not clearly defined. Lokhorst et al. demonstrated that approximately 50% of patients might respond to DLI with some patients achieving long-lasting remissions and potentially cure. However, a severe GvHD occurs in the majority of patients and the overall prognosis for allo-transplanted patients with relapsed MM treated with DLI remains rather poor [64]. However, it has been shown that the graft-versus-myeloma (GvM) effect in some cases occurs without GVHD, which might indicate that responses against tumor-specific antigens, as for instance mHags, are involved [64]. More recently, it was suggested that the clinical benefit of DLI might be higher in patients who receive DLI preemptively for treatment of residual disease compared to patients treated at the time of relapse [65]. Interestingly, both T and NK cells are considered major players in the GvM effect, whereas only T-cells are associated with alloreactivity against healthy tissues and GvHD [66]. Thus, allogeneic NK-cells therapy represents an interesting approach that will be further discussed in the following section.

### 3.3. Adoptive Transfer of NK Cells

An alternative to adoptive T-cell therapies is the transfer of NK cells (Figure 2), which has a direct capacity of killing malignant cells in an antigen-independent manner [67]. There is evidence of NK-cell dysfunction in MM [68,69,70] which makes NK-cell treatment attractive because NK-cell therapy can, in contrast to T-cell-based therapies, be based on an allogeneic source. Moreover, the use of a haploidentical allogeneic source might be therapeutically beneficial by inducing alloreactivity specifically against malignant cells without significant risk of GvHD [71,72]. NK-cell activity is regulated by a complex interplay of activating and inhibitory signals originating from target cells. Inhibition is provided by killer immunoglobulin-like receptors (KIRs) that recognize allotypic determinants displayed by different human leukocyte antigen (HLA) class I alleles. Inhibitory signals are generally dominant and prevent NK cells from killing autologous cells. Importantly, the number of HLA class I KIR ligands varies among individuals [73]. Therefore, transplantation across histocompatibility barriers may trigger donor NK-cell alloreactivity if the recipient lacks KIR ligands present in the donor. It was demonstrated that NK cells from KIR-ligand mismatched donors exert a potent anti-leukemic effect and prevent relapse after haploidentical transplantation for acute myeloid leukemia (AML) [72]. The potential curative effect of KIR ligand mismatched NK-cell therapies in AML has also been reported by others [74]. In MM, it has been suggested that the GvM effect in an allo-SCT setting is partially mediated by NK cells [75]. However, only a few studies in MM have used allogeneic KIR ligand-mismatched NK cells from haploidentical family donors. This approach has led to in vivo expansion of donor NK cells, but unconvincing response rates [71]. More recently, umbilical cord blood-derived NK cells have also been used in combination with autologous HSCT in heavily pretreated and high cytogenetic risk MM and the results from a phase 1 study encouraged a currently ongoing phase 2 study [76,77].

The use of allogeneic NK cells as immunotherapy for MM remains a promising concept. Importantly, the efficacy of existing MM therapies, including the IMIDs and monoclonal antibodies, relies substantially on endogenous NK cells [78,79,80]. Because endogenous NK cells are known to be functionally deficient in MM and may even interplay negatively with CD38 targeting therapies [81], there is a rationale for approaches where allogeneic NK cells are used in combination with already approved therapies (Figure 2). Recently, CD38 knockout (CD38^KO^) NK cells from healthy donors were generated. When compared to paired CD38 wild-type NK cells, CD38^KO^ NK cells showed less fratricide and superior persistence in mice in the presence of the CD38 antibody daratumumab (DARA). Additionally, CD38^KO^ NK cells exhibited enhanced ADCC against an MM cell line and primary MM cells from a patient with disease relapse on DARA [82]. Thus, using CD38^KO^ cells has the potential to maximize the clinical efficacy of DARA against MM and this concept is currently being evaluated in a phase 1 clinical trial (NCT04614636).

In the era of adoptive immune cell therapy when genetically modified cells are often used, the fact that therapeutic NK cells might originate from an allogeneic source, without inducing GvHD in the recipient, is currently its biggest advantage over T-cell-based approaches. However, many other differences between NK- and T-cell therapies exist, which originate from their role in the immune system, and both T-cell and NK-cell-based therapies might find their specific applications in different clinical scenarios in the near future. Adoptive transfer of genetically modified immune cells is discussed in the following section.

## 4. Adaptive Immune Transfer of Autologous and Allogeneic Lymphocytes with Genetic Manipulation (TCR–Engineered T cells, Chimeric Antigen Receptor T-Cells and Chimeric Antigen Receptor NK-Cells)

**Figure 3:**
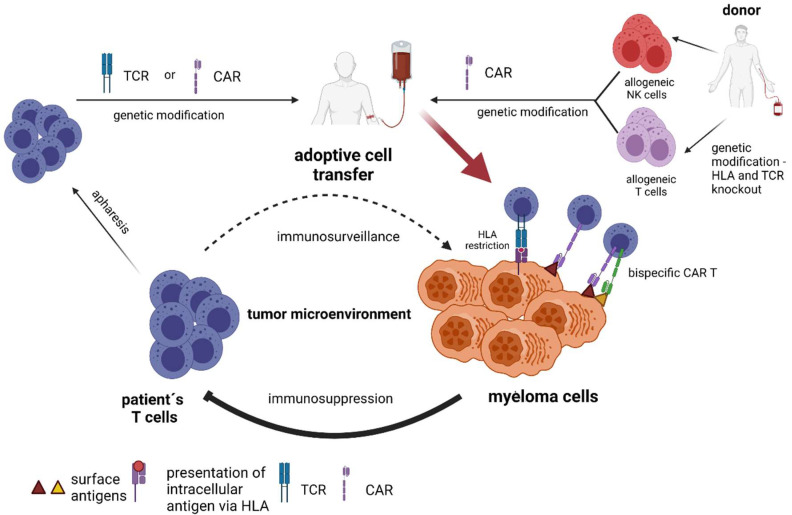
Adoptive immune transfer of autologous and allogeneic lymphocytes with genetic manipulation (TCR-engineered T cells, chimeric antigen receptor T-cells, and chimeric antigen receptor NK-cells).

The unique targeting specificity of immune cells can be re-directed by the integration of genes encoding either conventional alpha-beta T-cell receptors (TCR) or chimeric antigen receptors (CAR) (Figure 3). CARs are constructed by linking the antigen recognizing part of an antibody (scFv) to activating intracellular signaling molecules, often including costimulatory domains encoding CD3z, CD28, or CD137 to fully activate T-cells [83]. Many different CAR designs and approaches have been developed and are reviewed elsewhere [84]. Several methods to introduce CAR genes into immune cells with high efficiency exist, however, methods depending on viral vectors are clinically most developed and routinely used in FDA/EMA-approved products [85]. The importance of choosing a safe method for gene transfer is highlighted by a recent reporting of development of CAR T-cell-derived lymphoma in two of ten patients effectively treated with piggyBac modified CD19-specific CAR T-cells [86].

Perhaps the most important consideration confronting the use of genetically engineered cells is the selection of appropriate antigenic specificity of the introduced TCRs or CARs. The potency of T-cells enables the recognition of low levels of antigen expression on normal cells, which has led to severe on-target, off-tumor toxicity in patients [83]. Therefore, suitable antigen targets are those presented exclusively on the cancer or, alternatively, on normal cells that are not essential for survival [83]. Early demonstration of the power of T-cell therapy was demonstrated in melanoma patients who received high-avidity TCRs that recognized melanoma-melanocyte antigens. Although objective cancer regressions were observed, severe off-tumor/on-target toxicity was seen in the skin, eyes, and ears of patients due to the presence of melanocytes in these organs [87]. The same pitfalls of life-threatening on-target, off-tumor toxicities also apply to the use of CARs. A recent, and MM-relevant, demonstration of risk posed by targeting antigens broadly expressed in non-tumor tissues might be represented by CAR T-cells against CD38. While CD38 is broadly expressed in vital tissues, such as the liver and lung smooth muscle cells [88], the target is still considered a safe target for therapy with monoclonal antibodies [80]. However, a recent report on treatment with high-affinity CD38-specific CAR T-cells describes lethal adverse events observed after a patient received the anti-CD38 CAR T-cell infusion [89].

### 4.1. TCR–Engineered T Cells

The TCR recognizes peptides that are bound to HLA molecules on the cell surface. The peptides may be derived from both intracellularly and extracellularly expressed protein antigens. The first phase 1 trial of TCR-engineered T-cells in MM targeted the intracellular NY-ESO-1 antigen in HLA A*02:01-positive patients after auto-SCT. In this trial, TCR-engineered T-cells were trafficked to the marrow and showed extended persistence that correlated with encouraging clinical activity against antigen-positive myeloma [90]. Recently, the first two MM patients were treated with CRISPR-modified NY-ESO-1 specific TCR-engineered T-cells. In this study, genes for the PD-1 end endogenous TCR receptor were knocked out to improve T-cell function and persistence [91]. This pilot study demonstrated the safety and feasibility of multiplex CRISPR-Cas9 T-cell human genome engineering in patients with advanced MM, however, the conclusion in regards to efficacy is immature. Importantly, TCR-engineered T-cells can only be used in HLA-compatible patients whose malignant cells also express the targeted protein, thus their applicability is limited.

### 4.2. CAR-Engineered T-Cells

Compared to TCR-engineered T-cells, the introduction of CARs to T-cells provides non-HLA-restricted recognition of cell surface antigens, which significantly increases the spectrum of patients who may be treated. Unfortunately, a limited number of known cell surface antigens are uniformly expressed on plasma cells and not in other vital tissues, and this represents one of the greatest challenges in the development of MM-specific CAR T-cell therapies. Many extracellular proteins that are considered plasma cell-specific in regards to flowcytometric identification are also expressed in peripheral tissues (e.g., CD38, CD56, CD138) [89,92,93]. Targeting them thus represents a significant risk for on-target off-tumor toxicities. Interestingly, strategies tuning the antigen affinity of CAR T-cells have been described to discriminate healthy and tumor tissues based on their level of antigen expression. Drent et al. demonstrated that CD38-specific CAR T-cells with a reduced affinity could effectively lyse MM cells with a high CD38 expression but spare CD38 low healthy hematopoietic cells in vitro and in vivo [94]. This approach was suggested to be suitable for the generation of optimal CD38-specific CAR T-cells, which would spare healthy tissues and thus extend a spectrum of potentially targetable antigens. However, antigen downregulation is a common escape mechanism in tumors and might represent a major limitation of this approach [92].

### 4.3. Anti-BCMA CAR T-Cell Therapy

Currently, the most prominent example of an antigen targeted by CAR T-cells in MM is the B-cell maturation antigen (BCMA). BCMA is consistently expressed on the surface of normal and malignant plasma cells, thus by mature B cells. BCMA has a crucial role in the survival of long-lived plasma cells in the bone marrow and is not expressed in other vital tissues [93]. There are several BCMA CAR T-cell products in clinical development whose differences and clinical activity are reviewed elsewhere [95,96]. So far, only one of them, idecabtagene vicleucel, has received FDA approval based on recently published results from a phase 2 study (NCT03361748) [97,98], and ciltacabtagene autoleucel has earned FDA priority review [99], both for the indication of relapsed/refractory MM. Several clinical trials of anti-BCMA CAR T-cells have shown high-quality responses, including minimal residual disease negativity in patients with heavily pretreated multiple myeloma. Phase 3 trials are currently evaluating anti-BCMA CAR T-cell therapy versus standard-of-care regimens in patients in earlier stages of the disease and even in newly diagnosed patients with high-risk cytogenetic profiles or with residual disease after transplantation [95].

### 4.4. Resistance to Anti-BCMA CAR T-Cell Therapy

Although most MM patients initially respond to CAR T-cell treatment, only a subset of them have sustained responses for more than 1 year, and almost all patients eventually relapse. Relapses following CAR T-cell treatment are often related to the gradual loss of CAR T-cells, loss of antigen expression on the tumor cell surface known as antigen escape [95,100,101], or to an impaired T-cell activity induced by the immunosuppressive tumor microenvironment [102]. Several strategies to improve the effectiveness of CAR T-cell therapy are under investigation. These include optimizing CAR design and adapting the manufacturing process to generate cell products enriched for subsets of less differentiated T-cells [103]. The potential risk of relapse due to antigen escape and intratumoral heterogeneity with BCMA-negative subclones underscore the importance of targeting additional multiple myeloma-associated cell surface antigens. Several CAR T-cell products specific for other antigens, such as CD19, CD38, CD138, CD229, SLAMF7, and GPRC5D, are currently being evaluated in preclinical or clinical studies, and are reviewed elsewhere [95,96]. In addition, dual-antigen targeting combining BCMA with some alternative targets in bispecific CAR constructs proved an ability to prevent antigen escape in a mouse model [104,105]. An alternative approach of targeting two antigens simultaneously is represented by ligand-based CAR T-cells, which is based on ligand binding to two different receptors expressed on MM. APRIL-specific CAR T-cells allow for bispecific targeting of the MM-associated antigens BCMA and transmembrane activator and CAML interactor (TACI) which are natural receptors for APRIL ligand and are both uniformly expressed by MM [106]. Several phase 1 studies testing various designs of bispecific CAR T-cells and APRIL-based CAR T-cells are currently ongoing and reviewed elsewhere [98].

### 4.5. Allogeneic CAR T-Cells and CAR NK-Cells as ‘off the Shelf’ Adoptive Therapy

Using allogeneic CAR T-cells from donors (Figure 3) has many potential advantages over autologous approaches. CAR T-cells manufactured from healthy T-cells which were not exposed to previous lines of therapy and to immunosuppressive tumor microenvironment lead to better CART-cell proliferation and in vivo survival [107], which could, in turn, translate into long-lasting responses. In addition, allogeneic T-cells from donors are immediately available and provide a possibility for standardization of the CAR T-cell manufacturing with time for multiple cell modifications, which might decrease costs by using an industrialized process [108]. Conversely, allogeneic CAR T-cells may cause life-threatening GvHD or may be rapidly eliminated by the host immune system [108]. Nevertheless, rapid progress in gene-editing technologies has already resulted in strategies to control the risk of GvHD by efficiently eliminating TCR and HLA expression and have brought new techniques to make allogeneic CAR T-cells invisible to the host immune system [108]. There are currently several phase 1 studies testing anti-BCMA and anti-SLAMF7 allogeneic CAR T-cells in MM (NCT04093596, NCT04244656, NCT04171843, NCT04142619).

An alternative allogeneic source of immune cells that could be equipped with the CAR to kill MM cells is NK cells. Recently, allogeneic KIR mismatched, anti-CD19 CAR NK-cells derived from cord blood have shown to be an effective strategy to treat lymphoma patients [109]; however, there is currently only one running clinical trial investigating CAR NK-cells in MM (NCT03940833) [110]. Most of the ongoing CAR NK-cells trials in malignancies other than MM are testing CAR NK-cell products that are based on original CAR constructs tailored for T-cell activation, which may not be the best way to activate NK-cells. New CAR constructs with different activation domains might thus better reflect NK-cell biology and are in preclinical development. It is conceivable that they will pave the way for the successful development of CAR NK-cell therapy in the near future [111].

Rational combinations of CAR therapies with IMIDs, CD38 antibodies, and checkpoint inhibitors will likely enhance the performance of CAR therapy and will be discussed in the following section.

## 5. Strategies Mitigating Immune Resistance—Reviving of Existing Immune Responses and Stimulating Innate Immune System (Standard of Care Treatments, Immune Checkpoints Inhibitors, Bispecific Antibodies)

### 5.1. Standard of Care Treatments

**Figure 4:**
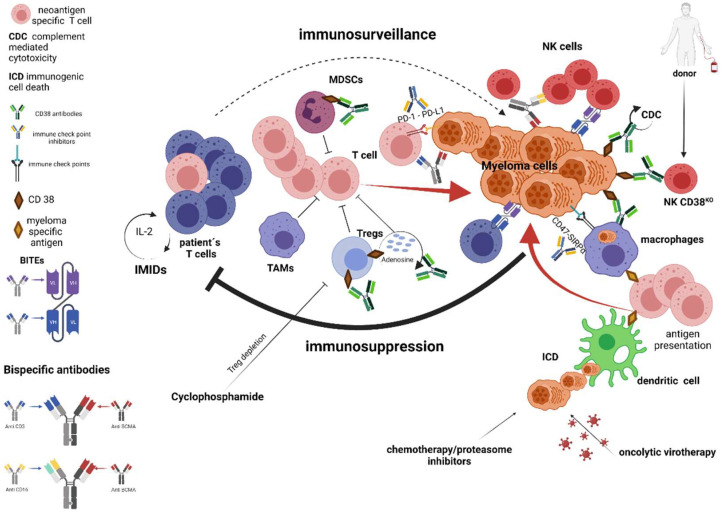
Strategies mitigating immune resistance—reviving of existing immune responses and stimulating innate immune system. ICD: Immunologic cell death; IMIDs: Immunomodulatory drugs; MILs: Marrow infiltrating lymphocytes; MDSCs: Myeloid-derived suppressor cells; TAMs: Tumor-associated macrophages, Tregs: Regulatory T cells.

The potential existence of myeloma-specific T-cell responses in MM patients is limited by the immunosuppressive TME. Strategies, which can mitigate such an immunosuppressive environment, or strategies that can revive and stimulate pre-existing immune responses, exist. Immunomodulatory drugs (IMIDs) are a cornerstone of MM treatment, and as their name suggests, immunomodulation is an important part of their mechanism of action mediated via IMIDs binding to cereblon, which induces enhanced affinity of cereblon to transcription factors IKZF1 and IKZF3. Subsequently, ubiquitination and degradation of these transcription factors lead to changes in gene transcription [112,113,114], including increased expression of IL-2, which regulates key aspects of T-cell biology. Via pathway signaling, IL-2 triggers critical metabolic and transcriptional changes that lead to T-cell survival, proliferation, and differentiation, which are critical for tumor control (Figure 4) [115].

Even classical chemotherapy might have a beneficial effect on immune-mediated anti-MM responses. Depletion of specific immunosuppressive populations has been described with cyclophosphamide treatment. The addition of low-dose cyclophosphamide to the IMID lenalidomide induced responses in lenalidomide refractory patients and a synergistic role of these two agents has been suggested [116]. It is well known that metronomic low-dose cyclophosphamide has multiple effects, including modulation of the microenvironment [117], and improvement of T- and NK-cell-mediated antitumor immune responses via depletion of regulatory T-cells [118,119,120,121]. It is unclear whether weekly higher-dose cyclophosphamide has the same effects on the bone marrow microenvironment compared with continuous low-dose cyclophosphamide (Figure 4) [116].

Certain anti-MM agents are able to provoke potent adaptive immune responses by inducing immunogenic cell death (ICD) [122] (Figure 4). Interestingly, there is a connection between endoplasmic reticulum (ER) stress and the occurrence of ICD [123]. MM cells display enhanced ER stress, hence making them dependent on ER stress-related survival pathways [123]. Compounds that target these survival pathways or that induce excessive amounts of ER stress are very effective in targeting MM cells, and it is tempting to speculate that ICD is an important mode of action of MM treatment as recently demonstrated for bortezomib [124]. Even radiation therapy might have an abscopal effect [125], which is a phenomenon of tumor regression in sites distant from targeted fields of irradiation and might be explained by the systemic immune response that radiation causes [126].

### 5.2. Monoclonal Antibodies Targeting CD38

Treatment with mAbs has recently become a milestone in the successful treatment of MM, outstandingly exemplified by daratumumab (DARA), a human CD38 mAb [80]. Treatment of MM with DARA is a passive immunization (Figure 4). After infusion, DARA binds to its target CD38 on MM cells, which in turn leads to activation of innate immune mechanisms (NK cells, complement system, and macrophages) to kill MM cells. It was demonstrated that high CD38 expression levels on the MM cell surface are important for effective killing by complement-mediated mechanisms and NK cells [127]. Nevertheless, DARA treatment leads to a reduction in CD38 levels on MM cells within a few hours after the first infusion [128,129]. Moreover, CD38-positive NK cells, the main effector cell population, are depleted by DARA-induced fratricide [81]. Therefore, it has been speculated how the downregulated CD38 expression is consistent with observed sustained responses to DARA treatment [130]. Recent data has suggested that CD38 metabolic pathways involving the production of immune-suppressive factor adenosine play an important role as a potential mechanism behind the suppression of adaptive immune responses [131,132]. It is therefore conceivable that the down-regulation of CD38 on both MM cells and cells of the tumor microenvironment by DARA treatment may lead to an improved adaptive immune response against MM cells [129]. In addition, DARA also eliminates CD38-positive immune suppressor cells, including regulatory T-cells, regulatory B cells, and myeloid-derived suppressor cells [133]. Altogether, DARA treatment results in a less immunosuppressive microenvironment, which explains why patients treated with this antibody experience clonal expansion of T-cells in both peripheral blood and bone marrow [133]. Importantly, the same induction of clonal T-cell expansion occurs with another CD38 mAb, isatuximab (ISA) [134] which coincides with an emerging adaptive immune response against myeloma-associated antigens NY-ESO-1 and CD38 [135], induced by ISA treatment. Interestingly, in DARA- and ISA-treated patients, the increase in T-cell clonality positively correlates with the response to treatment, suggesting that unique immunomodulatory characteristics of the CD38 target molecule and its downregulation by DARA mediated trogocytosis may actually be one of the explanations that CD38 antibodies show single-agent activity in MM [128,129]. These immunomodulatory properties of CD38 antibodies support the use of CD38 antibodies with other treatment modalities that enhance T-cell-mediated cytotoxicity as later discussed.

### 5.3. Immune Checkpoints Inhibitors

The discovery of immune checkpoint pathways, their role in the evasion from immune surveillance, and the development of blocking antibodies represent one of the greatest achievements in cancer treatment, which was also recently rewarded by the Nobel Prize [136]. The majority of studies in MM have focused on the inhibitory PD-1/PD-L1 pathway (Figure 4), but many more pathways are crucial for the function of the immune system and may be therapeutically manipulated [20]. The pre-clinical finding of high PD-L1 expression in malignant plasma cells, coupled with evidence suggesting a role for PD-L1 in the development of myeloma clonal resistance and relapse, drove the field towards pursuing immune checkpoint inhibitors in myeloma [137,138,139]. However, initial enthusiasm was followed by unsatisfying clinical results, and the potential role of PD-1/PD-L1 pathway inhibitors in MM is still a matter of debate. Based on preclinical data, IMIDs and DARA seemed to be ideal partners for the combination treatment [131,138,140,141] and early phase studies of the PD-1 inhibitor pembrolizumab, in combination with standard-of-care regimens for myeloma, showed promising results [142]. Nevertheless, excessive toxicity of the combination of pembrolizumab with lenalidomide or pomalidomide in phase 3 trials caused the FDA to suspend several trials in myeloma patients [143,144,145]. Combination studies of PD-1/PD-L1 pathway inhibitors with DARA have also been terminated based on results from a phase Ib/II LUC2001 study (NCT03023423). This study evaluated the efficacy and safety of a PD-L1 inhibitor, atezolizumab, with or without DARA in patients with advanced or metastatic non-small-cell lung cancer. No extra efficacy, but imbalanced death rates, were observed [141]. As a result, the sponsor stopped further enrollment on all studies with PD-1 or PD-L1 inhibitors combined with DARA [146].

Despite these disappointing results, the PD-1/PD-L1 pathway remains a viable treatment target in MM. Poor T-cell expansion and short-term T-cell persistence represent one of the main causes for the lack of response and relapse in therapies dependent on T-cell function (CAR T-cells, bispecific antibodies) [95]. It has been demonstrated that CAR T-cells from non-responders showed upregulated pathways involved in exhaustion and apoptosis [147]. Accordingly, the expression levels of T-cell co-inhibitory receptors in non-responders, such as PD-1, Tim-3, and LAG-3, were upregulated on CAR T-cells, suggesting possible inhibitory effects induced by these molecules [148,149]. Interestingly, PD-1 blockade resulted in clinical responses in a subset of patients with progression of B-cell lymphoma after anti-CD19-directed CAR T-cell therapy [148,149], and importantly, also in myeloma patients with a progression after anti-BCMA CAR T-cell therapy [150]. These observations demonstrated the proof of principle that inhibition of the PD-1/PD-L1 pathway can induce CAR T-cell re-expansion and responses in patients progressing after CAR T-cell treatment.

An “armored” CAR T-cell is among several approaches aiming to improve functions of engineered CAR T-cells by preventing T-cell exhaustion, enhancing killing function, and T-cell persistence [151]. Engineering CAR T-cells to secrete PD-1 or PD-L1 antibodies could prevent endogenous PD-1/PD-L1 axis activation in the TME, and thereby alleviate immune evasion [152,153]. An ongoing phase 1 clinical trial is exploring the safety of BCMA CAR T-cells secreting a mutant PD-1 Fc fusion protein (NCT04162119). Alternatively, the genome-editing technique may be used to silence PD-1 expression in T-cells and thus prevent T-cell exhaustion/activation cell death [91,154].

There are several clinical trials evaluating agents targeting other immune checkpoint pathways than the PD-L1/PD-1 pathway. LAG-3 and TIGIT antibodies are currently being evaluated as a monotherapy or in combination with standard treatments in MM (NCT04150965 and NCT04354246). Another promising immunotherapeutic target in MM is the macrophage immune checkpoint CD47 that provides a “do not eat me” anti-phagocytic signal. This molecule is overexpressed by virtually all cancers to enable the immune evasion of macrophages and other phagocytes [155]. CD47 overexpression is an independent predictor of a poor prognosis in patients with various cancer types, including hematological malignancies [21]. Anti-CD47 antibodies can induce phagocytosis of tumor cells by the blockade of CD47 and its ligand SIRPα [155,156]. In addition, CD47 antibodies induce an antitumor T-cell response by the cross-presentation of tumor antigens by phagocytes to T-cells (Figure 4) [157]. Thus, administration of CD47 antibodies together with an MM-targeting antibody such as DARA or elotuzumab might promote MM elimination in the same way as demonstrated in lymphoma patients treated simultaneously with rituximab and CD47 antibodies [155,158]. There are currently two clinical trials evaluating CD47 antibody (NCT04445701) and SIRPα-IgG4 Fc ligand (NCT03530683) as a monotherapy or in combination with bortezomib and dexamethasone in RRMM. Simultaneous targeting two different inhibitory pathways might be beneficial as demonstrated in the case of malignant melanoma [159].

### 5.4. Bispecific Monoclonal Antibodies

Bispecific monoclonal antibody (bsMAb) concomitantly binds to two different antigens, commonly the one being on T-cells and the other being an MM-associated antigen, thereby redirecting T-cells toward the MM cells (Figure 4). Thus, bsMAbs rely on the patient’s T-cells to elicit an antitumor response. The bsMAbs have the obvious advantage of being a true “off-the-shelf” product without the need for ex vivo genetic manipulation as seen with CAR T-cells [160]. Various bispecific antibody platforms are currently in clinical trials and technologies to build bsMAbs are reviewed elsewhere [161]. The majority of clinical trial data for bsMAbs in MM relates to the BiTE^®^ and DuoBody^®^ platforms. While a BiTE molecule consists of two different single-chain variable fragments (scFv), a DuoBody is a mAb with two different antigen-binding fragments (Fab) and a functional constant region fragment (Fc). The presence of the Fc domain can promote the stability of the molecule and increase the half-life time of DuoBodies [161]. BsMAbs in clinical development target the same spectrum of antigens as CAR T-cells treatment, with BCMA-targeting bsMAbs as the most advanced in terms of clinical testing. Nevertheless, clinical development of products targeting other antigens such as GPRC5D, CD38, or FcRH5 is ongoing [160]. Bispecific antibodies targeting other MM-associated antigens may be useful in patients with low baseline BCMA expression or heterogeneous BCMA expression. In addition, as in the case of CAR T-cells, treatment with bispecific antibodies may lead to antigen escape [162]. Thus, treatment with a bispecific antibody targeting another antigen may be more effective. In addition, a combination of bispecific antibodies, simultaneously targeting different antigens, may mitigate tumor antigen escape [163]. BCMA/CD3 BiTE AMG420 is the first-in-class bsMAb in MM. In a phase 1 trial, 70% of patients treated with the maximum tolerated dose achieved a partial remission or better, with minimal residual disease (MRD) negative CR in 50% of patients [162]. A major limitation of the early BiTE products, including AMG420, is their short half-life time, which requires continuous infusion in order to achieve a steady therapeutic plasma level. Several other BCMA/CD3 BiTEs or DuoBodies with an extended half-life time, such as AMG701, CC-93269, teclistamab, and REGN-5458, have since emerged and are under rapid clinical development. Their preliminary clinical characteristics are reviewed here [160]. Notably, most BCMA targeting BsMAbs show a relatively similar safety profile and efficacy in patients treated with the maximum tolerated dose. Dose expansion parts of these studies are currently ongoing, and phase 3 trials are upcoming.

Interestingly, in vitro experiments demonstrated that the direct combination of BCMA/CD3 DuoBody teclistamab with DARA enhances MM cell lysis in an additive fashion. Teclistamab-mediated tumor cell lysis was superior when T-cells obtained from patients treated with DARA were used, compared to T-cells from DARA naïve MM patients [164]. This positive effect of DARA, on the ability of teclistamab to kill MM cells, might be related to the immune-stimulating effects of DARA already mentioned [133]. These data formed a rationale for the first clinical trial investigating bsMAbs as part of a multi-targeted combination therapy. A phase 1 TRIMM study is evaluating the efficacy of teclistamab plus DARA in RRMM (NCT04108195, https://clinicaltrials.gov/ct2/show/NCT04108195) (accessed on 7 June 2021). IMiDs might also provide T-cell-stimulating effects and thus are a promising partner for bispecific antibodies. Indeed, pretreatment of effector cells with lenalidomide or pomalidomide enhanced AMG701-mediated lysis of MM cells [165]. As already mentioned, checkpoint signaling is an important immunosuppressive component of the myeloma microenvironment [138], which might limit the anti-myeloma activity of the bsMAbs. Importantly, it was shown that PD-L1 blockade may enhance bsMAbs-induced MM cell elimination in vitro [166]. Currently, several clinical studies are investigating the concept of bsMAbs/checkpoint inhibition combination therapies in B-cell malignancies (NCT02879695, https://clinicaltrials.gov/ct2/show/NCT02879695 (accessed on 7 June 2021), NCT03340766, https://clinicaltrials.gov/ct2/show/NCT03340766) (accessed on 7 June 2021), and promising first safety data have been reported for combining CD19/CD3 BiTE blinatumumab and PD-1 inhibitor pembrolizumab [167].

The first experience with bsMAbs suggested a lower response of these agents compared to CAR T-cell therapy. Interestingly, in contrast to CAR T-cells, bsMAbs do not provide a co-stimulatory T-cell signal via CD137 or CD28 molecules, which might induce T-cell anergy and thus compromise the efficacy of bsMAbs. In vitro data has shown that CD28 co-stimulation enhances the efficacy of CD19/CD3 bsMAbs, a finding reminiscent of data obtained with CAR T-cells when significant improvements in their potency were achieved after the inclusion of co-stimulatory signaling moieties in the CAR constructs [168]. Therefore, the concept of tri-specific antibody (tsMAb) has emerged. TsMAb targets three antigens: a cancer cell, a receptor that activates T- cells, and a T-cell protein that promotes long-lasting T-cell activity against the cancer cell [169]. SAR442257 represents a new tri-specific monoclonal antibody that binds to CD3 and CD28 on T-cells and CD38 expressed on MM cells. The resulting cross-linkage may trigger a potent cytotoxic T-lymphocyte (CTL) response against CD38-expressing tumor cells [169]. In addition, SAR442257 can also directly target CD28 expressed on MM cells, thereby enhancing the anti-tumor activity of this agent and allowing it to bind to tumor cells when CD38 is downregulated after treatment with CD38 antibodies [170]. SAR442257 is currently being investigated in a phase 1 study in RRMM (NCT04401020).

Recently, an interesting concept of on-target restoration of a split T-cell-engaging antibody has emerged. This approach is based on a T-cell-engaging antibody derivative that comes in two complementary halves called hemibodies and addresses antigen combinations instead of single molecules [171]. Two different hemibodies contain an antigen-specific scFv fused to the variable light or variable heavy chain domains of a CD3 antibody. When the two hemibodies simultaneously bind their respective antigens on a single cell, they align and reconstitute the original CD3-binding site to engage T-cells. In preclinical models, hemibodies could preferentially induce the cellular lysis of double-positive cells and spare single-positive cells [171].

More recently, the discovery of scFv that recognizes peptides derived from mutant p53 presented via the HLA class I molecule, provided a promising approach for targeting previously non-druggable intracellular targets presented by tumor cells at very low density [172]. It was demonstrated that bsMAbs comprising an scFv specific for mutant p53/HLA-A2 fused to an scFv specific for CD3 activated with high sensitivity T-cells in vitro to lyse MM cells expressing less than 10 copies of p53/HLA-A2 complexes per cell. Moreover, immunodeficient mice engrafted with human T-cells suppressed the growth of a human MM cell line expressing an average of 2.4 copies of p53/HLA-A2 per cell after treatment with the p53/CD3 bsMAbs [173]. Unfortunately, the polymorphic nature of classical HLA class I limits the individuals to be targeted with this approach. To broaden population coverage, an alternative may be to find neoantigen peptides presented by non-classical HLA (that is, HLA-E and HLA-G). Unlike classical HLA, HLA-E and HLA-G are essentially monomorphic, and their preponderance is up-regulated on cancer cells. Whether HLA-E and HLA-G present peptides from shared MM neoantigens may be worth investigating [172].

BsMAbs and tsMAbs engaging NK cells are also being explored in preclinical and clinical studies. RO7297089 (AFM26), a BCMA/CD16A-directed bsMAb induced NK cell-mediated lysis of BCMA-positive myeloma cell lines in vitro and is currently being investigated in a phase 1 study (NCT04434469) [174]. Although still in the preclinical stage of development, trispecific NK cell engager showed promising in vitro results. TsMAb targeting CD16A and the MM antigens BCMA and CD200 induced enhanced cytotoxicity of BCMA and CD200 double-positive myeloma cells compared with single-positive cells [175].

## 6. Oncolytic Virotherapy

The oncolytic potential of viruses was first recognized almost four decades ago when a series of case studies documented the regression of various hematological malignancies in patients with concurrent measles infections [176,177] Oncolytic virotherapy yields results from the direct killing of malignant cells by oncolytic virus replication in cancer cells. Interestingly, MM-specific mutations in the RAS pathway [178] and overexpression of some cell-surface proteins that are commonly used as viral entry receptors render MM cells sensitive to viral infection [179,180,181]. Moreover, the oncolytic death of infected MM cells is immunogenic and promotes cross-presentation of released MM proteins by tumor-resident antigen-presenting cells, and thereby also enhancing antitumor adaptive T-cell responses (Figure 4) [182]. Oncolytic viruses are derived from naturally occurring strains that can be further genetically modified to increase their specificity to MM cells and enhance their ability to promote tumor clearance. Several oncolytic viruses that have been proposed as therapeutics in MM are reviewed elsewhere [180,181]. The most prominent example is a measles virus (MV-NIS) engineered to express the human thyroidal sodium iodide symporter (NIS). The NIS construct allows for the concentration of radioiodine in tumor cells, which can be utilized both for noninvasive in vivo visualization of virus-infected cells using single-photon emission computed tomography or in therapeutic synergy with β-emitting radioiodine [183,184]. It has been demonstrated that MV-NIS could induce significant tumor regression in myeloma xenografts, as well as in MV-resistant MM tumors when combined with I^131^ radiotherapy [183]. MV-NIS uses CD46 receptors to enter MM and to drive intercellular fusion of infected cells with their uninfected neighbors, resulting in the formation of nonviable multinucleated syncytia [183]. Myeloma plasma cells overexpress CD46 and are therefore highly susceptible to MV-NIS killing [179]. In a completed phase 1 dose-escalation study, only 1 out of 27 treated patients achieved complete remission, however, less durable decreases in myeloma-specific IgG and/or serum-free light chain (FLCs) levels were seen in other patients [185]. Only two additional patients were enrolled in a dose-expansion phase 2 study evaluating MV-NIS with cyclophosphamide in MM. Both patients passed away while enrolled, and the study has been terminated. Recently reported results of a phase 1 trial, NCT03017820, showed that systemic administration of genetically modified vesicular stomatitis virus (VSV) to patients with heavily pretreated hematological malignancies did not induce any objective responses in MM patients, however, responses have been observed in patients with T cell lymphoma and future trials of combination strategies with immune-modulatory drugs are currently being planned [186].

## 7. Conclusions

Immune dysfunction is a hallmark of MM and worsens by disease progression. Immunotherapy aims to overcome this immune dysfunction by harnessing the host immune system to treat MM, and several immunotherapeutic approaches are being developed and are undergoing current clinical testing (Figure 1). The ultimate goal is to cure MM, however, so far, only a few patients treated with allogeneic immune cells (allo-SCT) have achieved this, suggesting that autologous immune cells from MM patients might be incapable of achieving long-term MM control. Therefore, using an allogeneic source of immune cells for CAR T-cells or CAR NK-cells might represent a promising strategy. In addition, combination therapies of the new T-cell or NK-cell engaging approaches with immunomodulating agents are promising as the next wave of treatment. Neoantigen-specific antibodies/CAR T-cells could further widen the spectrum by targeting oncogenic driver mutations. Data from clinical trials testing all these new approaches and their combinations are eagerly awaited.
